# Peer Review of “Performance Drift in Machine Learning Models for Cardiac Surgery Risk Prediction: Retrospective Analysis”

**DOI:** 10.2196/60280

**Published:** 2024-06-12

**Authors:** Juntong Zeng

**Affiliations:** 1 Fuwai Hospital, National Center for Cardiovascular Diseases, Chinese Academy of Medical Sciences and Peking Union Medical College, Beijing, China

**Keywords:** cardiac surgery, artificial intelligence, risk prediction, machine learning, operative mortality, data set drift, performance drift, national data set, adult, data, cardiac, surgery, cardiology, heart, risk, prediction, United Kingdom, mortality, performance, model

*This is the peer-review report for “Performance Drift in Machine Learning Models for Cardiac Surgery Risk Prediction: Retrospective Analysis.”*

## Round 1 Review

### General Comments

This manuscript [1] presents an interesting study that explores temporal trends in various performance metrics for different types of prediction models used in the prediction of in-hospital mortality after cardiac surgery in the United Kingdom from 2012 to 2019. The data set was divided into 2 periods: from 2012 to 2016 for model training and internal validation and from 2017 to 2019 for external validation. The study evaluated 5 prediction models: logistic regression, support vector machine (SVM), random forest, extreme gradient boosting (XGBoost), neural network, and European System for Cardiac Operative Risk Evaluation (EuroSCORE) II. The authors aimed to assess the model performance on 5 metrics (1 – expected calibration error [ECE], area under the curve [AUC], 1 – Brier score, *F*_1_-score, and net benefit) and proposed a composite metric, the clinical effectiveness metric (CEM), calculated as the geometric mean of the 5 mentioned metrics, as the primary metric.

The study began with a nontemporal baseline evaluation of different models in the 2017‐2019 temporal validation and then conducted a series of drift analyses, including an examination of overall trends from 2012 to 2019, within-period trends in the first 3 months of 2017 and 2019, and between-period trends between the first 3 months of 2017 and 2019. The authors also analyzed drift in variable importance and variable distribution, defined by the temporal change in the ratio of several top-importance features within the data set, to profile data set drift.

The authors demonstrated that XGBoost and random forest were the best-performing models, both in nontemporal and temporal evaluations, whereas the EuroSCORE II model exhibited a significant drop in performance. Temporal declines in model performance were observed across all models and were consistent with data set drift.

Overall, the question of the generalizability of prediction models, whether temporal or spatial, has long been a topic of discussion in clinical research. This study takes a commendable approach to addressing this question. However, there are some issues that require clarification and revision, including (1) methodological concerns related to the justification of the main metric (CEM) using averaging, and the appropriateness of some statistical tests; (2) the clinical significance of the identified performance drift; and (3) the overall clarity of the study’s design and presentation.

### Specific Comments

#### Major Comments

1. The statement of the study’s objectives should be improved for more clarity, particularly regarding the phrase “verify suspected dataset drift by assessing the relationship between and within performance drift, variable importance drift, and dataset drift across ML and ES II approaches.” It is unclear what is meant by the “relationship between and within.” Does this refer to the analysis of performance drift within and between different periods? The overall study design is quite challenging to grasp initially, even with the graphical overview provided in Figure 1. To enhance clarity, additional details and explanations should be added to the aims, overall design, graphical overview, and text the *Methods* and *Results* sections.

2. The rationale for introducing CEM as the primary performance metric, calculated as the geometric mean of 5 distinct individual metrics, is debatable and lacks strong justification. Although the geometric mean is less sensitive to outliers compared to the arithmetic mean, it raises the fundamental question of why these metrics need to be summarized. Is it merely to obtain a single quantitative measure for analysis, or does it aim to provide a more comprehensive understanding of overall model performance? It appears to serve primarily the former purpose, which may not be an appropriate practice given that the 5 metrics assess entirely different aspects of model performance: 1 – ECE for calibration, AUC for discrimination, 1 – Brier score (which already encompasses calibration and discrimination components), *F*_1_-score for threshold-specific discrimination, and net benefit index for cost-effectiveness. Consequently, interpreting the exact meaning of CEM becomes challenging, as it reduces these diverse aspects to a single numerical value. Therefore, I suggest just reporting and examining all 5 metrics individually, with or without highlighting certain ones as primary areas of interest.

3. The manuscript used several statistical tests, and some of them are relatively less commonly used. Please provide a more detailed description of the objectives and specific statistical situations for each test used. Additionally, for the baseline nontemporal performance comparison, a more conventional approach for comparing AUC would be the use of the DeLong method (you could choose the best model as the reference), and bootstrapping can be used to assess the statistical significance when comparing other metrics.

4. During the training and internal validation phase with 5-fold cross-validation, additional details are needed to understand how the final model for each model type was selected for subsequent temporal validation, including whether hyperparameter tuning was carried out and whether there was a final refitting process on the entire training data set following the cross-validation, etc.

5. The *Introduction* section should incorporate more background information on previous studies reporting or relating to performance variation in prediction models for cardiac surgery outcomes. In the *Discussion* section, it is also important to discuss how this work contributes to existing evidence in the context of these previous studies. Some relevant studies, based on my preliminary search, include Benedetto et al [2], Zeng et al [3], Mori et al [4], and potentially more.

6. Although the authors observed numerical declines in CEM and other metrics, the magnitude of these declines appears to be relatively small, particularly when considering metrics such as AUC. As a result, it is essential to discuss how to interpret this magnitude of drift in the context of clinical practice. In other words, what is the clinical significance of this variation in performance, and how does it justify the necessity of actively monitoring model drift in terms of cost-effectiveness? Please discuss.

7. The conclusion should only focus on the primary findings outlined in the aims of the *Introduction* section. Avoid incorporating less central findings and speculative elements. Additionally, it may not be fair to suggest replacing the EuroSCORE II model simply based on the inferior performance in this study, since it was already established and this study essentially conducted an external validation for it, whereas the other machine learning models were developed using these data sets.

#### Minor Comments

1. More detailed definitions and explanations should be provided for each performance metric.

2. In the *Methods* section, please provide a clear outline of the inclusion and exclusion criteria. Additionally, consider including a flowchart that illustrates the data set development process, outlining how these criteria were applied.

3. I had difficulty understanding what “outliers” and “distribution” meant in the *Results* section for the baseline nontemporal performance of each model. I thought that each metric of each model should be just a numerical value and a 95% CI from bootstrapping.

4. The title of the manuscript should be an objective reflection of the overall study design and aim, rather than drawing conclusions from the findings.

5. I did not find the supplementary materials in the review system. I am not sure whether this issue is on my end or not.

## Round 2 Review

### General Comments

I appreciate the opportunity to rereview this manuscript. The authors’ efforts in revising their manuscript in response to previous concerns are commendable. This manuscript has been improved and is now in principle publishable. It could potentially be accepted upon reasonable response to a few follow-up minor comments, outlined below.

### Specific Comments

1. About my previous major comment 1, the authors meticulously elaborated on (1) the reasons for performance drift and (2) its importance, which are both valid points. However, the current *Introduction* (lines 121‐179) is quite lengthy. I recommend consolidating these 2 parts into a single paragraph, listing each point without the need for detailed individual explanations. Additionally, my query about the exact meaning of “the relationship between and within variable importance drift, performance drift, and actual dataset drift” remains unaddressed. Even though it was removed from the *Introduction*, it still appears in the abstract. I suggest the authors explicitly explain it to readers and incorporate it into the manuscript when first mentioned.

2. Regarding the justification for the CEM, the authors have added more explanation and supporting literature for its use. However, it would strengthen their case if they could provide examples from external studies or use cases where a similar practice (averaging different aspects of metrics for model performance evaluation) was used, beyond their own studies.

3. About the statistical tests for comparing AUC with the DeLong method, I believe that performing the DeLong test for AUC comparison is not overly computationally demanding, even on a relatively large data set. I recommend the authors explore commonly used R packages (eg, “pROC”) that facilitate AUC calculation and comparison with the DeLong method. The DeLong comparison typically requires paired variables of the label and 2 models’ predicted probabilities, and the 95% CI and *P* value are automatically calculated by bootstrapping these paired samples, which is relatively efficient.

4. Regarding model tuning and specification of the best models (PS: I still cannot find the supplements, only a revised clean manuscript; I am not sure if this was due to issues from my end), I am curious why different tuning practices were used for different models, especially grid search for XGBoost and SVM but manual tuning for random forest.

5. In response to the query about the clinical significance of the relatively small scale of performance drift, the authors referred to one of their previous studies briefly discussing this matter. However, it would be much clearer if the authors could more explicitly elaborate in this study and, if possible, provide additional analysis to support this argument.

## Abbreviations

AUC: area under the curve
CEM: clinical effectiveness metric
ECE: expected calibration error
EuroSCORE: European System for Cardiac Operative Risk Evaluation
SVM: support vector machine
XGBoost: extreme gradient boosting
